# Pentadecanoic acid promotes basal and insulin-stimulated glucose uptake in C2C12 myotubes

**DOI:** 10.29219/fnr.v65.4527

**Published:** 2021-01-22

**Authors:** Wen-Cheng Fu, Hai-Yan Li, Tian-Tian Li, Kuo Yang, Jia-Xiang Chen, Si-Jia Wang, Chun-Hui Liu, Wen Zhang

**Affiliations:** 1 School of Life Sciences, East China Normal University, Shanghai, China; 2 China National Institute of Standardization, Beijing, China

**Keywords:** pentadecanoic acid, glucose uptake, AMP-activated protein kinase, insulin sensitivity, C2C12 myotubes

## Abstract

**Background:**

Saturated fatty acids (SFAs) generally have been thought to worsen insulin-resistance and increase the risk of developing type 2 diabetes mellitus (T2DM). Recently, accumulating evidence has revealed that SFAs are not a single homogeneous group, instead different SFAs are associated with T2DM in opposing directions. Pentadecanoic acid (C15:0, PA) is directly correlated with dairy products, and a negative association between circulating PA and metabolic disease risk was observed in epidemiological studies. Therefore, the role of PA in human health needs to be reinforced. Whether PA has a direct benefit on glucose metabolism and insulin sensitivity needs further investigation.

**Objective:**

The present study aimed to investigate the effect and potential mechanism of action of PA on basal and insulin stimulated glucose uptake in C2C12 myotubes.

**Methods:**

Glucose uptake was determined using a 2-(N-[7-nitrobenz-2-oxa-1,3-diazol-4-yl] amino)-2-deoxyglucose (2-NBDG) uptake assay. Cell membrane proteins were isolated and glucose transporter 4 (GLUT4) protein was detected by western blotting to examine the translocation of GLUT4 to the plasma membrane. The phosphorylation levels of proteins involved in the insulin and 5’-adenosine monophosphate-activated protein kinase (AMPK) pathways were examined by western blotting.

**Results:**

We found that PA significantly promoted glucose uptake and GLUT4 translocation to the plasma membrane. PA had no effect on the insulin-dependent pathway involving insulin receptor substrate 1 (Tyr632) and protein kinase B (PKB/Akt), but increased phosphorylation of AMPK and Akt substrate of 160 kDa (AS160). Compound C (an AMPK inhibitor) blocked PA-induced AMPK activation and reversed PA-induced GLUT4 translocation, indicating that PA promotes glucose uptake via the AMPK pathway *in vitro*. Moreover, PA significantly promoted insulin-stimulated glucose uptake in myotubes. Under insulin stimulation, PA did not affect the insulin-dependent pathway, but still activated AMPK.

**Conclusion:**

PA, an odd-chain SFA, significantly stimulates glucose uptake via the AMPK-AS160 pathway and exhibits an insulin-sensitizing effect in myotubes.

## Popular scientific summary

- Epidemiological findings have shown a negative association between circulating pentadecanoic acid (C15:0) and metabolic disease risk.
- Pentadecanoic acid, an odd-chain saturated fatty acid, significantly stimulated basal glucose uptake via the AMPK-AS160 pathway in myotubes, indicating it has a direct benefit on glucose metabolism.
- Pentadecanoic acid significantly enhanced insulin-stimulated glucose uptake, exhibiting an insulin-sensitizing effect.

Obesity, especially abdominal obesity, is an important determinant of the risk for developing insulin resistance and non-insulin-dependent diabetes mellitus (1). It can be induced by dietary habits, as high fat intake may increase the risk of obesity (1, 2). Moreover, it is suggested that a high proportion of fat in the diet is associated with impaired insulin sensitivity and an increased risk of developing diabetes, independent of obesity and body fat localization; moreover, this risk may be influenced by the type of fatty acids in the diet (1). Generally, saturated fatty acids (SFAs) have been thought to worsen insulin-resistance and increase the risk of developing type 2 diabetes mellitus (T2DM), while monounsaturated and polyunsaturated fatty acids improve these features (2).

Recently, accumulating evidence has revealed that SFAs are not a single homogeneous group, instead different SFAs are associated with T2DM in opposing directions (3). A number of studies concluded that tissue concentrations of even-chain SFAs are linked to metabolic dysfunction, while circulating even-chain fatty acids are positively associated with the risk of T2DM (4). However, higher proportions of pentadecanoic acid (15:0, PA) and heptadecanoic acid (17:0) in erythrocyte membranes were found to be associated with a lower risk of diabetes (5). Moreover, a negative association between metabolic disease risk and circulating odd-chain fatty acids, such as PA and heptadecanoic acid, has been reported (6, 7). A large prospective case-cohort study (3) that assessed plasma levels of individual SFAs and new-onset T2DM also revealed a positive association between even-chain SFAs (14:0, 16:0, and 18:0) and the incidence of T2DM, whereas odd-chain SFAs (15:0 and 17:0) were inversely associated with this disease. These findings emphasize the importance of recognizing SFA subtypes. Historically, odd-chain SFAs were used as internal standards for gas chromatography-mass spectrometry of total fatty acids and liquid chromatography-mass spectrometry of intact lipids, as it was thought that their concentrations were insignificant in humans (6). Following these important observations, the role of odd-chain SFAs (especially C15:0 and C17:0) in human health needs to be reinforced.

Through combinations of both animal and human intervention studies, Benjamin and colleagues investigated all possible contributions of odd-chain SFAs from the gut microbiota, diet, and novel endogenous biosynthesis. They found that circulating C15:0 and C17:0 are independently derived; C15:0 correlates directly with dietary intake, while C17:0 is substantially biosynthesized (7).

When dietary correlates were assessed, odd-chain SFAs were associated most strongly with dairy products (4, 5). Pentadecanoic acid is directly correlated with dairy products, and a negative association between circulating PA and metabolic disease risk was observed in epidemiological studies (3). Previous studies reported that dairy fat might reduce insulin resistance and T2DM (5). However, whether PA has direct physiological benefits on insulin resistance and T2DM, or is merely correlated with other beneficial compounds in dairy fat needs further investigation. Glucose uptake by peripheral tissues, such as skeletal muscles and adipocytes, is important for the maintenance of glucose homeostasis (8), and serves as a mechanism for the prevention or amelioration of hyperglycemia and T2DM. Skeletal muscle is responsible for approximately 75% of glucose uptake, which is regulated by two distinct pathways: one stimulated by insulin through insulin receptor substrate 1 (IRS1)/phosphoinositide 3 kinase (PI3K) (9), and another stimulated by muscle contraction and exercise through activation of AMPK (10).

Therefore, the present study investigated the effect of PA on glucose uptake in C2C12 myotubes and its potential mechanism of action by focusing on insulin and/or AMPK signaling pathways mediating glucose uptake.

## Materials and methods

### Chemicals

We purchased PA from Chengdu Huaxia Reagent (Sichuan, China). Compound C was obtained from Selleck Chemicals (Shanghai, China). A glucose oxidase determination kit was purchased from Shanghai Kexin Biotechnology Research Institute (Shanghai, China). Insulin was from Novo Nordisk (Shanghai, China). A β-actin antibody was purchased from Shanghai Yeasen Biotech (Shanghai, China). Phospho-Akt (Thr308), phospho-Akt (Ser473), AS160, and phospho-AS160 (Thr642) antibodies were purchased from Cell Signaling Technology (Danvers, Massachusetts, USA). Phospho-IRS1 (Tyr632) and GLUT4 antibodies were purchased from Abcam (Cambridge, UK). IRS1, Akt, and ATPase Na^+^/K^+^ Transporting Subunit Alpha 1 antibodies were purchased from Proteintech (Wuhan, China). An AMPKα antibody was purchased from Abgent (San Diego, California, USA), while a phospho-AMPKα (Thr183/Thr172) antibody was obtained from Arigo Biolaboratories Corp. (Taiwan, China).

### Cell culture and differentiation

The C2C12 mouse myoblasts were obtained from The National Center for Drug Screening (Shanghai, China). C2C12 mouse myoblasts were maintained in Dulbecco’s Modified Eagle’s Medium (DMEM) supplemented with 10% (v/v) fetal bovine serum, streptomycin (100 U/mL), and penicillin (100 U/mL) at 37°C with 5% CO_2_. Cells were seeded into culture plates at a density of 5 × 10^4^ cells/mL. After 24 h (about 70% confluence), the medium was switched to DMEM supplemented with 2% (v/v) horse serum, which was replaced after 2, 4, and 6 days of culture. After 6–7 days, differentiation was complete.

### MTT assay

The effect of PA on cell viability was measured using an 3-(4,5-dimethylthiazol-2-yl)-2,5-diphenyltetrazolium bromide (MTT) assay. Briefly, C2C12 mouse myoblasts were cultured in 96-well plates. After differentiation, cells were incubated in DMEM containing 0.2% bovine serum albumin (BSA) for 6 h. The culture medium was then changed to DMEM containing 0.2% BSA and various concentrations of PA for 48 h. Following incubation, 20 μL of 3 mg/mL MTT was added to each well and incubated for 2.5 h at 37°C. Next, 200 μL of dimethyl sulfoxide was added to each well and oscillated until MTT formazan crystals dissolved. Finally, the absorbance of each well was quantified at 490 nm using a microplate spectrophotometer, and cell viability was calculated according to the following formula:

$$Cell\;viability\%=\frac{\text{ODsample}-\text{ODblank}}{\text{ODcontrol}-\text{ODblank}}\times 100\%$$

### Glucose consumption assay

Glucose consumption from the culture media was determined using a glucose assay kit. Briefly, after differentiation, C2C12 myotubes were incubated in DMEM containing 0.2% BSA for 6 h and then treated with different doses of PA for 12, 24, and 48 h. After treatment, glucose concentrations in the medium were measured using a commercially available glucose oxidase assay kit according to the manufacturer’s protocol. The amount of glucose consumed was calculated by dividing the glucose concentration in cell-plated wells from values observed in blank wells.

### 2-NBDG uptake assay

Cell glucose uptake was determined by measuring 2-(N-[7-nitrobenz-2-oxa-1,3-diazol-4-yl] amino)-2-deoxyglucose (2-NBDG) uptake according to the following procedure. Mature C2C12 mouse myotubes were cultured in black 96-well plates and treated with varying concentrations of PA for a range of time periods. At 1 h before harvest, cells were washed twice with warm sterile phosphate-buffered saline (PBS), and then the medium was switched to glucose-free DMEM supplemented with 0.2% BSA. After 1 h, cells were washed once with warm sterile PBS and then incubated with the same medium containing 80 μM 2-NBDG for 30 min. Cells were then washed once more with warm sterile PBS before measuring the fluorescence intensity of each well (485-nm excitation and 520-nm emission). Cell glucose uptake was calculated according to the following formula:

$$\text{Cell glucose uptake}=\frac{FI_{Sample}-FI_{Blank}}{FI_{Control}-FI_{Blank}}$$

### Western blotting analysis

After treatments, cells were washed twice with ice-cold PBS and then harvested in radioimmunoprecipitation assay lysis buffer (150 mM sodium chloride, 1.0% Triton X-100, 0.5% sodium deoxycholate, 0.1% sodium dodecyl sulfate, and 50 mmol/L Tris; pH 8.0) containing protease and phosphatase inhibitors. Cell membrane proteins were extracted using a Mem-PERa Plus Membrane Protein Extraction Kit (Thermo Fisher Scientific, Waltham, Massachusetts, USA) according to the manufacturer’s protocol. Protein concentrations were determined using a Bicinchoninic acid Protein Assay Kit (Yeasen Biotech, Shanghai, China) according to the manufacturer’s protocol. Equal quantities of protein were separated on 10% sodium dodecyl sulfate-polyacrylamide gels and transferred to nitrocellulose membranes, which were incubated overnight at 4°C with primary antibodies. After three washes in Tris-buffered saline containing 0.1% Tween-20, membranes were incubated for 1 h with secondary antibodies at room temperature. Finally, blots were washed and visualized on an Odyssey CLx Imaging System (LI-COR Biosciences, Lincoln, Nebraska, USA), and then analyzed by Image-Pro Plus Software (Media Cybernetics, Rockville, Maryland, USA).

### Statistical analysis

Data are expressed as mean ± standard deviation (SD). Statistically significant differences among experimental groups were determined by one-way analysis of variance followed by Dunnett’s multiple-comparisons tests using SPSS software (IBM, Armonk, New York, USA). We considered *P*-values of less than 0.05 and 0.01 to indicate significant and extremely significant differences, respectively.

## Results

### Pentadecanoic acid enhanced glucose uptake and GLUT4 translocation in C2C12 myotubes

**Fig. 1:**
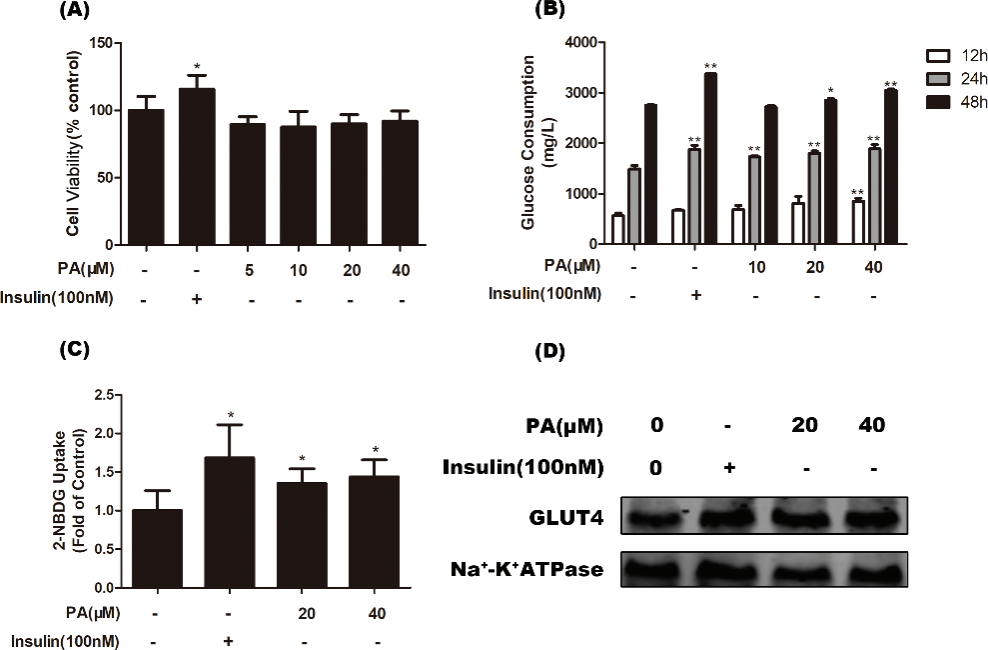
Effects of PA on glucose uptake and GLUT4 translocation in C2C12 myotubes. (a) Cells were incubated with PA (0–40 μM) or insulin (100 nM) for 48 h, and viability was determined by MTT assay (*n* = 6). (b) Myotubes were treated with various doses of PA for 12, 24, or 48 h, and glucose consumption was assayed by a commercially available glucose oxidase assay kit (*n* = 4). (c) After incubating C2C12 myotubes with PA (20 and 40 μM) for 24 h or insulin (100 nM) for 30 min, glucose uptake was measured using the 2-NBDG method (*n* = 5). (d) Cells were treated with PA or insulin (100 nM) for 24 h, and then western blotting of GLUT4 in cell membrane protein fractions was performed. Values are expressed as mean ± SD. ^*^ *P* < 0.05, ^**^ *P* < 0.01, versus control group.

The potential cytotoxicity of PA on C2C12 myotubes was evaluated by MTT assay. As shown in Fig.1a, treatment of cells with 5, 10, 20, or 40 μM PA for 48 h did not obviously affect viability. Therefore, PA at concentrations less than 40 μM were used in subsequent experiments.

We first evaluated the effects of PA on glucose consumption and uptake in C2C12 myotubes. As shown in Fig. 1b, PA significantly increased glucose consumption of myotubes. Treatment of cells with 40 μM PA for 12, 24, and 48 h increased glucose consumption compared with control cells (*P* < 0.01). Moreover, a 2-NBDG uptake assay was used to determine the effect of PA on glucose uptake. The results showed that 20 and 40 μM PA significantly enhanced glucose uptake in C2C12 myotubes by 35.1 and 43.8%, respectively, compared with controls (*P* < 0.05, Fig. 1c). Furthermore, we evaluated the effect of PA on GLUT4 translocation to the plasma membrane. Consistent with the results of glucose uptake, GLUT4 levels in the plasma membrane were also increased by PA (Fig. 1d).

### Characterization of signaling pathways involved in pentadecanoic acid-induced glucose uptake and GLUT4 translocation in C2C12 myotubes

To explore the underlying molecular mechanism by which PA promotes cell glucose uptake and GLUT4 translocation, phosphorylation levels of proteins involved in insulin and AMPK pathways were examined using western blotting analysis.

**Fig. 2:**
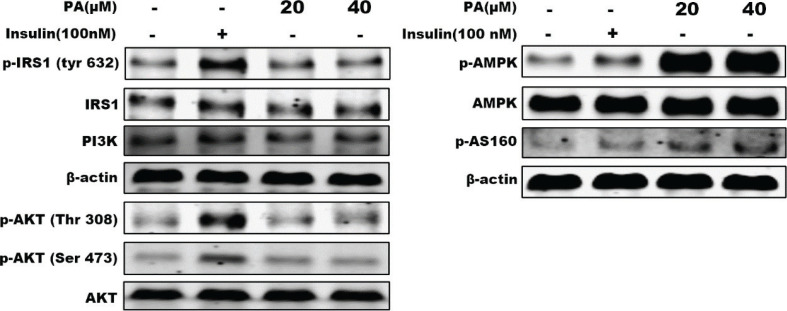
Effects of PA on proteins involved in insulin and AMPK signaling pathways as shown by western blotting of whole cell lysates from C2C12 myotubes incubated with PA (20 or 40 μM) for 24 h. Protein levels of PI3K and phosphorylation (p) of four proteins were examined: IRS1, Akt, AMPK, and AS160. β-actin was used as a standard.

The insulin signaling pathway plays an important role in glucose uptake and GLUT4 translocation. To evaluate the effects of PA on the insulin signaling pathway in the present study, activation of IRS1 and Akt were measured using western blotting. Results shown in Fig. 2a indicate that treatment with PA (20 or 40 μM) for 24 h had no effect on phosphorylation levels of IRS1 (Tyr632) or Akt (Ser473 and Thr308), indicating that PA enhanced translocation of GLUT4 through another signaling pathway.

In addition to insulin signaling, the AMPK pathway plays an important role in GLUT4 translocation and glucose uptake. Thus, we further evaluated the contribution of AMPK to PA-stimulated glucose uptake in the present study. Activation of AMPK and its downstream target AS160 were assayed. As shown in Fig. 2b, PA markedly increased phosphorylated levels of AMPK (Thr172) and AS160 in C2C12 myotubes, suggesting that the effects of PA on glucose uptake and GLUT4 translocation were not related to the insulin signaling pathway, but rather the AMPK pathway.

### Pentadecanoic acid enhanced glucose uptake and GLUT4 translocation through the AMPK pathway

**Fig. 3:**
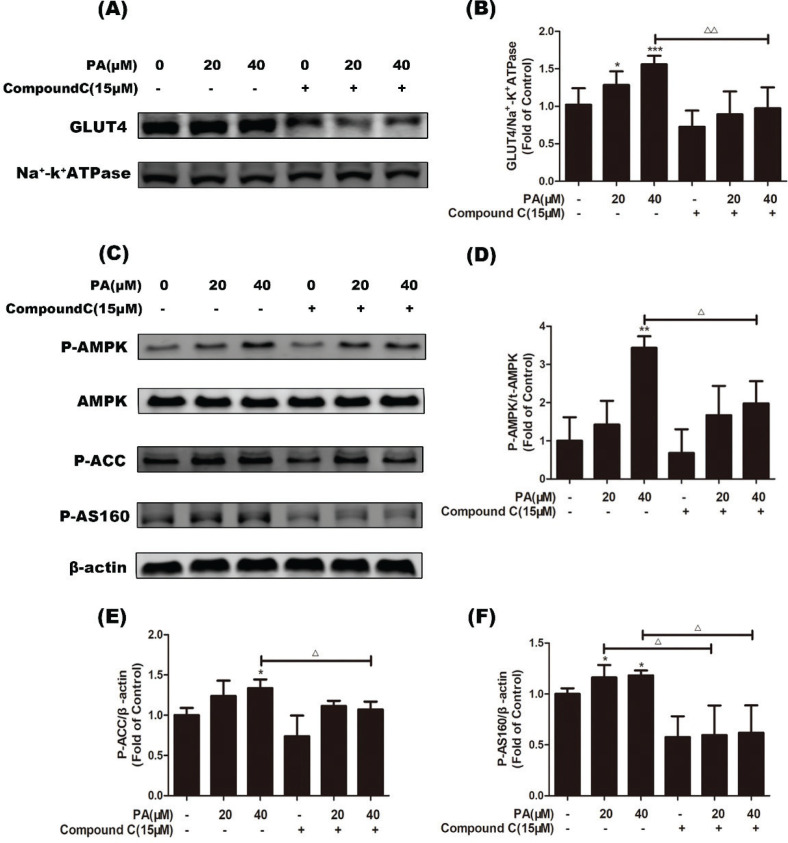
Effects of the AMPK inhibitor compound C on PA-induced GLUT4 translocation and AMPK activation. C2C12 myotubes were incubated in the presence or absence of compound C (15 µM) for 1 h followed by exposure to PA for 24 h, and then GLUT4 in cell membrane proteins was detected. Western blotting (a) and quantification (b) of GLUT4 in cell membrane protein fractions (*n* = 4). Cells were incubated in the presence or absence of compound C (15 µM) for 1 h followed by exposure to PA for 6 h. Western blotting of whole cell lysates was performed to detect phosphorylation of AMPK, ACC, and AS160. Western blotting (c) and quantification (d–f) of phospho-AMPK, phospho-ACC, and phospho-AS160 (*n* = 3). Values are expressed as mean ± SD. ^*^ *P* < 0.05, ^**^ *P* < 0.01, ^***^ *P* < 0.001, versus control group; ^Δ^ *P* < 0.05, ^ΔΔ^ *P* < 0.01, versus PA group.

To confirm whether the promotional effect of PA on GLUT4 translocation was mediated through AMPK activation, we pretreated myotubes with the AMPK-specific inhibitor compound C (15 μM, 1 h), followed by treatment with PA (20 and 40 μM). As shown in Fig. 3a and b, PA-induced GLUT4 translocation was decreased in myotubes pretreated with compound C (*P* < 0.01 vs. PA treatment alone). Furthermore, compound C blocked the upregulation of phosphorylation of AMPK, ACC, and AS160 induced by PA (Fig. 3c–f, *P* < 0.05 vs. PA treatment alone). These results indicated that PA exerts a beneficial effect on glucose uptake based on GLUT4 translocation in C2C12 myotubes via the AMPK pathway.

### Pentadecanoic acid ameliorated insulin-stimulated glucose uptake and GLUT4 translocation in C2C12 myotubes

**Fig. 4:**
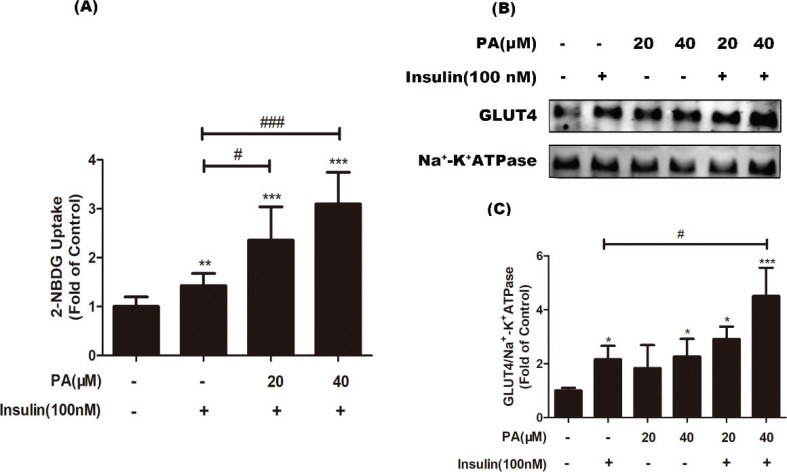
Effects of PA on insulin-stimulated glucose uptake in C2C12 myotubes. (a) Cells were incubated with PA (20 and 40 µM) for 24 h, followed by exposure to insulin (100 nM) for 30 min. Next, glucose uptake was measured by a 2-NBDG method (*n* = 6). (b) Myotubes were treated with PA, insulin, or PA and insulin for 24 h before western blotting of GLUT4 in cell membrane proteins was performed (*n* = 3 or 4). (c) Quantification of GLUT4. Values are expressed as mean ± SD. ^*^ *P* < 0.05, ^**^ *P* < 0.01, ^***^ *P* < 0.001, versus control group; ^#^ *P* < 0.05, ^##^ *P* < 0.01, ^###^ *P* < 0.001, versus insulin group.

It is known that some long-chain fatty acids, such as palmitic acid and stearic acid, inhibit insulin-stimulated glucose uptake. To investigate whether PA has an inhibitory effect on insulin-induced glucose utilization, we evaluated the effect on insulin-stimulated glucose uptake. C2C12 cells were treated with 100 nM insulin in the presence or absence of PA, and a 2-NBDG assay was performed. As shown in Fig. 4a, in the presence of insulin, PA (20 or 40 μM) significantly improved insulin-induced glucose uptake by 1.65- and 2.17-fold, respectively, compared with insulin treatment alone (*P* < 0.05 or *P* < 0.01). To confirm this observation, GLUT4 translocation to the plasma membrane was detected by western blotting. When PA was co-administered with insulin for 24 h, GLUT4 translocation was also further increased (*P* < 0.05 vs. insulin treatment alone, Fig. 4b and c), indicating PA significantly enhanced insulin-induced GLUT4 translocation. This result, which was consistent with the promoting effect of PA on glucose uptake, indicated the ability of PA to promote insulin-mediated glucose utilization. Thus, PA has an insulin-sensitizing effect in myotubes.

### Upon stimulation by insulin, pentadecanoic acid had no effect on insulin signal transduction, but activated AMPK

**Fig. 5:**
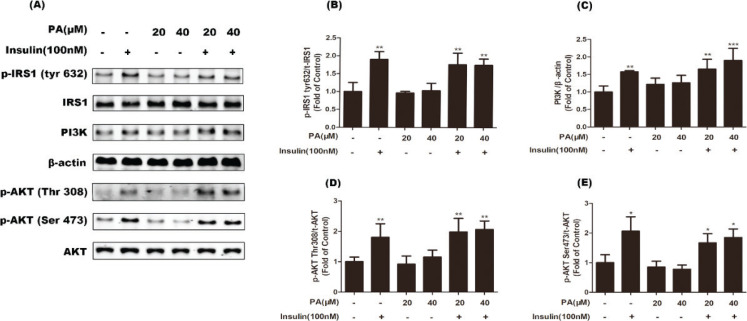
Effects of PA on insulin-stimulated phosphorylation of IRS1 and Akt, and protein expression of PI3K in C2C12 myotubes. Cells were incubated with PA (20 and 40 µM), insulin (100 nM), or PA and insulin for 24 h. Western blotting of whole cell lysates to detect phosphorylation (p) of IRS1 and Akt, and protein expression of PI3K. Western blotting (a) and quantification (b–e) of phospho-IRS1, phospho-Akt, and PI3K (*n* = 3 or 4). Values are expressed as mean ± SD. ^*^ *P* < 0.05, ^**^ *P* < 0.01, versus control group.

Insulin obviously enhanced phosphorylation of IRS1 (Tyr632) and Akt (Thr308, Ser473), and increased protein expression of PI3K (Fig. 5). Co-treatment with PA for 24 h did not affect insulin-stimulated activation of the IRS/PI3K/Akt pathway (Fig. 5). These results suggest that PA improved insulin-induced glucose uptake through another pathway independent of insulin.

**Fig. 6:**
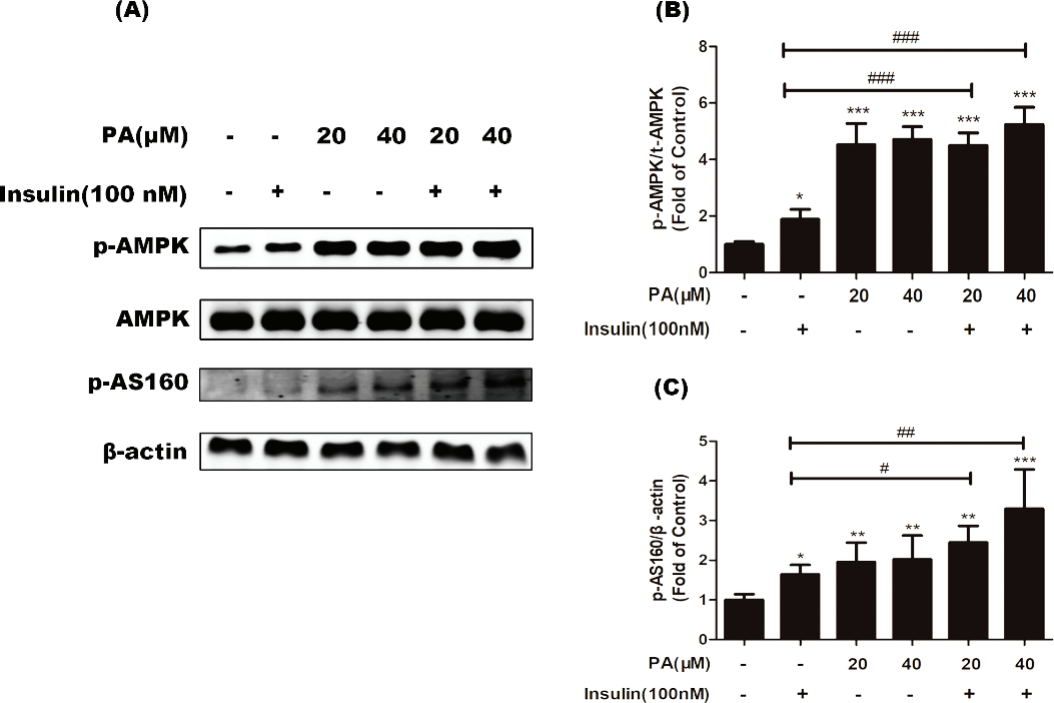
Effects of PA on phosphorylation of AMPK and AS160 under insulin stimulation in C2C12 myotubes. Cells were incubated with PA (20 and 40 µM), insulin (100 nM), or PA and insulin for 24 h. Western blotting of whole cell lysates to detect phosphorylation of AMPK and AS160. Western blotting (a) and quantification (b, c) of phospho-AMPK and phospho-AS160 (*n* = 3 or 4). Values are expressed as mean ± SD. ^*^ *P* < 0.05, ^**^ *P* < 0.01, versus control group; ^#^ *P* < 0.05, ^##^ *P* < 0.01, ^###^ *P* < 0.001, versus insulin group.

In addition to the insulin pathway, the AMPK pathway mediates GLUT4 translation. As shown in Fig. 6, co-treatment of myotubes with PA and insulin markedly increased the phosphorylation of AMPK (*P* < 0.01) and AS160 (*P* < 0.05 or *P* < 0.01) compared with cells treated with insulin alone. These results suggest that PA likely enhanced insulin-mediated glucose uptake via AMPK signaling.

## Discussion

Epidemiological studies have revealed a negative association between circulating PA (C15:0) and metabolic disease risk. In the present study, PA was found to have direct benefits on glucose metabolism, as it promoted GLUT4 translocation to the plasma membrane and subsequently stimulated glucose uptake through activation of the AMPK signaling pathway in C2C12 myotubes. Furthermore, PA promoted insulin sensitivity in C2C12 myotubes and significantly enhanced insulin-stimulated GLUT4 translocation and glucose uptake.

In skeletal muscle cells, GLUT4 translocation is central for glucose metabolism. Translocation of GLUT4 from intracellular vesicles to the plasma membrane is the most important step regulating glucose uptake, and can be promoted by activation of PI3K/Akt/AS160 and AMPK/AS160 pathways (11). In this study, treatment with PA significantly increased basal glucose uptake in myotubes. After isolating membrane proteins, GLUT4 translocation was detected. The results showed a significant increase of GLUT4 expression in membrane protein extracts of PA-treated cells, indicating that PA treatment stimulated GLUT4 translocation to the plasma membrane. This result was consistent with the stimulatory effect of PA on glucose uptake, indicating that PA enhanced GLUT4 translocation and subsequently stimulated glucose uptake.

Next, the signaling pathways involved in PA-caused glucose uptake and GLUT4 translocation were characterized. Our results demonstrated that PA significantly increased AS160 phosphorylation in C2C12 cells. AS160 is a Rab GTPase-activating protein comprising a Rab-GAP domain, two phosphotyrosine-binding domains, a calmodulin-binding domain, and five canonical Akt phosphomotifs (12). Convincing evidence implicates Akt2-dependent AS160 phosphorylation of Thr642 as a key part of the mechanism for insulin-stimulated glucose uptake by skeletal muscle (12). AS160 is targeted not only by Akt but also other kinases including AMPK and protein kinase C, leading to increased surface levels of GLUT4 (13). Treatment with PA for 24 h did not affect phosphorylated levels of IRS1 (Tyr632) or Akt (Ser473 and Thr308), indicating that PA had no effect on the insulin pathway at tested concentrations. These results suggest the promoting effect of PA on GLUT4 translocation was probably mediated through another signaling pathway. Next, we examined whether AMPK was involved in PA-induced glucose uptake. Pentadecanoic acid was able to activate AMPK in a dose-dependent manner, suggesting that AMPK activation was probably associated with PA-stimulated glucose uptake. Next, the AMPK inhibitor compound C was used to test this hypothesis. We observed that compound C blocked PA-induced phosphorylation of AMPK and its downstream targets ACC and AS160. In addition, PA-induced increases in GLUT4 translocation were abolished by compound C treatment, indicating that PA stimulated glucose transport via AMPK. Therefore, our results demonstrated that PA significantly stimulated basal glucose uptake in myotubes, exhibiting a direct benefit on glucose metabolism.

A large prospective case-cohort study (3) revealed that odd-chain SFAs (15:0 and 17:0) were inversely associated with T2DM, in contrast to even-chain SFAs. Based on epidemiologic and biological evaluations, it is generally accepted that even-chain SFAs induce insulin resistance (3, 14). Palmitic acid (16:0), a common dietary saturated free fatty acid, seems to be especially harmful in animal and *in vitro* models (4). Even at a low concentration (20 µM), palmitic acid can markedly induce insulin resistance in skeletal muscle cells (15). However, whether PA has a direct benefit on insulin sensitivity is still unclear. Thus, we examined whether PA affects insulin-mediated glucose regulation. To this end, C2C12 cells were treated with 100 nM insulin in the presence or absence of PA. When PA was co-treated with insulin, GLUT4 translocation was further increased. To confirm this observation, we performed a glucose uptake assay, and found that when PA was co-administered with insulin, glucose uptake was also further increased. These results indicated that co-treatment of PA and insulin improved insulin sensitivity.

A key action of insulin is the stimulation of glucose uptake into cells by inducing the translocation of GLUT4 from intracellular storage sites to the plasma membrane (16). Insulin signaling pathway mediates this process. Insulin binds to the insulin receptor, which activates its tyrosine kinase activity and subsequently the IRS/PI3K/Akt/AS160 pathway. Next, GLUT4 translocation is mediated by phosphorylation of AS160, which inhibits its Rab GTPase-activating protein activity (17). Palmitic acid increases phosphorylation of IRS1 serine residues concomitantly with decreased tyrosine phosphorylation of IRS1, resulting in attenuated downstream insulin signaling via IRS1 and Akt, thus reducing glucose uptake into the cell by GLUT4 and attenuating insulin-stimulated glucose uptake (15, 18–20).

Our data revealed that PA did not affect insulin-stimulated activation of IRS/PI3K/Akt pathway, which is different from the inhibitory effect of palmitic acid. In addition, when cells were co-treated with insulin, PA still strongly activated AMPK. As AMPK activation is known to increase insulin sensitivity (21), the enhancement of insulin-stimulated glucose uptake in myotubes by PA is likely associated with AMPK.

In summary, this pilot study showed that PA (C15:0) significantly stimulated basal glucose uptake and promoted insulin-induced glucose uptake in C2C12 myotubes. These findings provide a possible mechanism for the negative association between circulating pentadecanoic acid and metabolic disease risk.
